# Reduced growth rate of aged muscle stem cells is associated with impaired mechanosensitivity

**DOI:** 10.18632/aging.203830

**Published:** 2022-01-13

**Authors:** Mohammad Haroon, Heleen E. Boers, Astrid D. Bakker, Niek G.C. Bloks, Willem M.H. Hoogaars, Lorenzo Giordani, René J.P. Musters, Louise Deldicque, Katrien Koppo, Fabien Le Grand, Jenneke Klein-Nulend, Richard T. Jaspers

**Affiliations:** 1 Laboratory for Myology, Department of Human Movement Sciences, Faculty of Behavioural and Movement Sciences, Vrije Universiteit Amsterdam, Amsterdam Movement Sciences, Amsterdam 1081 HZ, The Netherlands; 2 Department of Oral Cell Biology, Academic Centre for Dentistry Amsterdam, University of Amsterdam and Vrije Universiteit Amsterdam, Amsterdam Movement Sciences, Amsterdam 1081 LA, The Netherlands; 3 Sorbonne Université, INSERM UMRS974, Center for Research in Myology, Paris 75013, France; 4 Department of Physiology, Amsterdam University Medical Center VUmc, Amsterdam Cardiovascular Sciences, Amsterdam 1081 HZ, The Netherlands; 5 Institute of Neuroscience, Université Catholique de Louvain, Louvain-la-Neuve 1348, Belgium; 6 Exercise Physiology Research Group, Department of Movement Sciences, KU Leuven, Leuven 3001, Belgium; 7 Faculty of Medicine and Pharmacy, NeuroMyoGène UCBL-CNRS UMR 5310, INSERM U1217, Lyon 69008, France

**Keywords:** aging, mechanosensitivity, muscle stem cell, proliferation, YAP signaling

## Abstract

Aging-associated muscle wasting and impaired regeneration are caused by deficiencies in muscle stem cell (MuSC) number and function. We postulated that aged MuSCs are intrinsically impaired in their responsiveness to omnipresent mechanical cues through alterations in MuSC morphology, mechanical properties, and number of integrins, culminating in impaired proliferative capacity. Here we show that aged MuSCs exhibited significantly lower growth rate and reduced integrin-α7 expression as well as lower number of phospho-paxillin clusters than young MuSCs. Moreover, aged MuSCs were less firmly attached to matrigel-coated glass substrates compared to young MuSCs, as 43% of the cells detached in response to pulsating fluid shear stress (1 Pa). YAP nuclear localization was 59% higher than in young MuSCs, yet YAP target genes *Cyr61* and *Ctgf* were substantially downregulated. When subjected to pulsating fluid shear stress, aged MuSCs exhibited reduced upregulation of proliferation-related genes. Together these results indicate that aged MuSCs exhibit impaired mechanosensitivity and growth potential, accompanied by altered morphology and mechanical properties as well as reduced integrin-α7 expression. Aging-associated impaired muscle regenerative capacity and muscle wasting is likely due to aging-induced intrinsic MuSC alterations and dysfunctional mechanosensitivity.

## INTRODUCTION

Aging-associated reduction in muscle regeneration after injury and loss of muscle mass is referred to as sarcopenia [1, 2]. Prime mechanistic factors underlying the loss of muscle mass are myofiber atrophy and loss of myofibers [2]. This myofiber loss is attributed to an impaired regenerative capacity of aged muscle and associated muscle stem cells (MuSCs) [3]. Muscle regeneration relies on the proper functioning of myofibers and their associated MuSCs, which are self-renewing skeletal muscle precursor cells involved in muscle growth, repair, and regeneration [4, 5]. MuSCs differentiate along the myogenic lineage establishing a transitory population of proliferating cells known as myoblasts, which then fuse with growing myofibers and provide them with nuclei [6]. During aging, MuSCs lose their potential to regenerate the damaged myofiber [7], resulting in an imbalance between muscle degeneration and regeneration leading to a loss of muscle mass [8]. The mechanism of reduced MuSC function with ageing is still not fully understood.

Several mechanisms have been proposed to contribute to impaired MuSC function, in particular alterations in the MuSC niche and cell-intrinsic changes [9, 10]. MuSC function and muscle regeneration are facilitated by a multitude of soluble biochemical factors secreted by macrophages, fibroblasts, and the host myofiber [11]. These biochemical signals are disrupted with age [12, 13]. Moreover, parabiosis experiments have shown that aged serum is detrimental to MuSC function [12, 14]. Conversely, MuSCs from aged mice (aged MuSCs) grafted onto young muscles are deficient in self-renewal and regenerative capacity [15]. This agrees with the fact that aged MuSCs acquire cell-intrinsic alterations during aging, and that these are not reversed by altering the niche conditions [13, 15]. In other words, exposure to young niche conditions is not sufficient to rejuvenate aged MuSCs. This raises the question of which other, possibly cell-intrinsic, mechanisms are involved in MuSC dysfunction.

MuSCs in their niche are anchored to the sarcolemma of the host myofiber, i.e., on their basal side via cadherins, and on their apical side to the basal lamina via integrins, syndecans, and dystroglycans [16]. Upon myofiber stretch-shortening, MuSCs are subjected to mechanical loads due to tensile and shear deformations of the extracellular matrix (ECM; i.e., endomysium) [17, 18]. These deformations also cause interstitial fluid movement within the muscle [19]. Currently it is unknown whether MuSCs can sense these fluid movements. Differentiated myotubes respond to shear forces applied as fluid shear stress by upregulation of nitric oxide (NO), interleukin-6 (*IL-6*), and cyclooxygenase-2 (*COX2*) [20]. NO is known to play a role in MuSC activation and muscle regeneration [21]. MuSCs are known to respond to tensile forces and alterations in ECM mechanical properties [22, 23], but whether the response of MuSCs and differentiated myotubes to shear forces is similar is currently unknown. The mechanical loads are transmitted through molecular complexes connecting cells with the extracellular environment, such as integrins clustered in focal adhesions, that trigger the expression of growth factors and cytokines regulating MuSC function [11], thereby contributing to the prevention of muscle wasting. Aging is known to be associated with a decline in integrin number and/or function in MuSCs, possibly due to increased fibrosis and ECM stiffness [12, 24, 25]. However, whether this decline persists when the aged MuSCs are subjected to a different substrate outside their niche, and whether this is accompanied by changes in mechanosensitivity is unknown [26].

The intracellular domain of integrins attaches to various proteins to form focal adhesions, which connect to the cytoskeleton [27]. Thus, integrins mediate the interactions between the cell and their niche, and link the cytoskeleton to the ECM. In response to biochemical and mechanical stimuli, integrins regulate cell shape [28], initiate signaling pathways [29], and alter gene expression [30]. Integrin clustering signals focal adhesion kinase (FAK) recruitment and its association with either integrin-β subunit or paxillin to initiate downstream signaling [31]. FAK activation increases yes-associated protein (YAP) activity and nuclear localization [32]. Stiff substrates cause increased FAK activation, enhanced stress fiber formation, cell spreading, and elevated YAP activity/nuclear localization [32–34]. This leads to increased cell proliferation and survival [34–36]. Moreover, mechanical forces acting on the cell also regulate YAP activity and nuclear translocation, which contributes to ECM remodeling [34, 37]. The hippo pathway and its effector YAP modulate the mechanical properties of the cell as well as cell adhesion by regulating expression of focal adhesion genes, thus affecting focal adhesion formation and sensing ECM stiffness [38]. YAP also plays a role in skeletal muscle regeneration by affecting MuSC self-renewal [36]. Activated YAP binds to several transcription factors within the nucleus including DNA binding transcription factors, i.e., TEA domain family member 1 TEAD1, TEAD2, TEAD3, and TEAD4 [39]. The TEAD family of transcription factors is essential for YAP transcriptional activity and its function in cell proliferation, regeneration, and stem cell maintenance and differentiation [40]. YAP activation enhances TEAD target gene expression, i.e., *Ctgf* and *Cyr61*, and promotes cell proliferation and migration [35, 41]. It has been suggested that cell volume is also correlated to nuclear YAP levels [42]. Whether YAP nuclear translocation and transcriptional activity are dysregulated in aged MuSCs is hereto unknown, but YAP nuclearization/activity could be an indicator of an altered capability for mechanosensing in MuSCs. The consequences of impaired mechanosensitivity of aged MuSCs could be extensive since mechanical cues are ever present, whether they are derived from muscle activity or niche conditions, and have a paramount effect on cell function. For example, cellular sensing of substrate stiffness affects stem cell adhesion, morphology, self-renewal, and fate decisions [43, 44]. Whether adhesion, morphology, focal adhesions, YAP nuclearization/activity, and proliferation, are intrinsically altered in aged MuSCs has not yet been investigated, but if the ability for mechanosensing is deteriorated in aged MuSCs, these parameters are bound to be affected as well.

This study aimed to elucidate whether aged MuSCs are intrinsically impaired in their ability to sense and respond to mechanical cues. Such a disturbed ability for mechanosensing in aged MuSCs would lead to alterations in growth rate, focal adhesion number, as well as nuclear translocation of YAP, compared to young MuSCs. Furthermore, differences in cell shape and mechanical properties are strong indicators of altered capability for mechanosensing. Here we investigated aged MuSC growth rate, shape, and expression of proliferation- and myogenic-related genes, i.e. cyclin D1 (*Ccnd1*), cyclin-dependent kinase 4 (*Cdk4*), cyclin-dependent kinase inhibitor 2A (*Cdkn2a*), *Yap*, *Pax7*, *MyoD*, *Myog* (myogenin), as well as expression of YAP-binding partners, i.e. *Tead1*, *Tead2*, *Tead3*, and *Tead4*, compared to young MuSCs *in vitro*. We further explored the MuSC response to shear forces applied as fluid shear stress by measuring NO production and changes in expression of genes crucial for MuSC proliferation and regenerative function. Moreover, fluid shear stress-induced MuSC detachment was investigated followed by comparing the integrin-α7 (ITGA7) and phospho-paxillin (pPXN) clusters as a read-out of MuSC adhesion strength, focal adhesion number, and size. YAP cellular localization and expression levels of YAP-regulated genes were determined to measure the effect of age and mechanical loading on cellular functions regulated by YAP.

## RESULTS

### Aged muscle stem cells exhibit reduced growth rate

**Figure 1:**
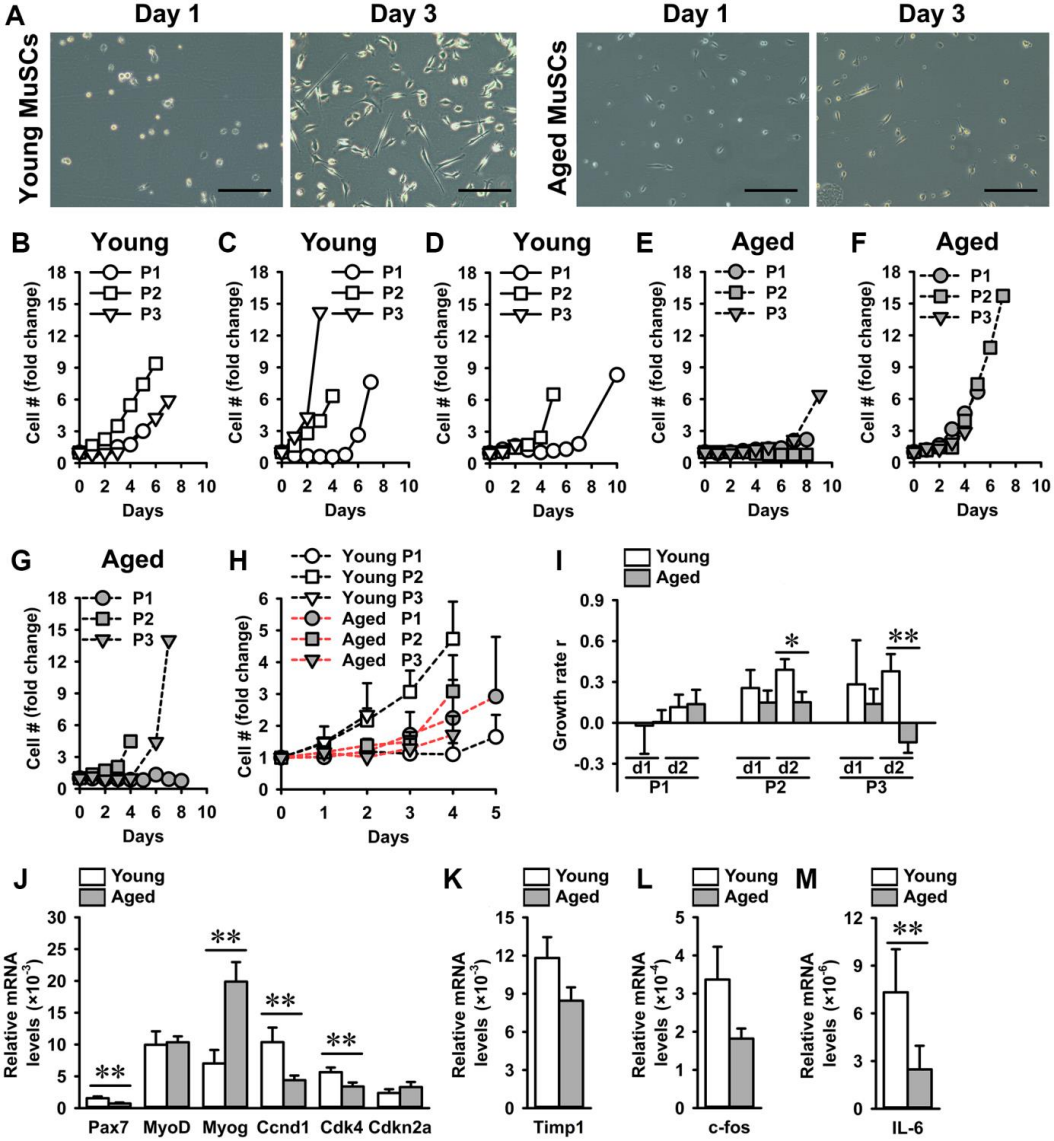
**Aged MuSCs exhibited a reduced growth rate compared to young MuSCs.** (**A**) Micrographs of young and aged MuSCs at day 1 and 3 of culture. (**B**–**G**) Fold-change in cell number of young and aged MuSCs *in vitro* at three passages (P1-P3). Each graph shows young and aged MuSCs, *n* = 1 (from 1 young or aged mouse). (**H**) Fold-change in cell number of young and aged MuSCs at three passages (P1-P3; pooled data), over the initial cell number seeded, as a function of time (days). *n* = 3 (from 3 young or aged mice). (**I**) Growth rate r of young and aged MuSCs at three passages (P1-P3) over two days illustrating an increased growth rate of young MuSCs at P2 and P3. *n* = 3 (from 3 young or aged mice). (**J**–**M**) Gene expression of *Pax7*, *MyoD*, *Myog*, *Ccnd1*, *Cdk4*, *Cdkn2a*, *Timp1*, *c-fos*, and *IL-6*. Young MuSCs, *n* = 11 (from 4 young mice). Aged MuSCs, *n* = 9 (from 3 aged mice). Abbreviation: MuSCs: muscle stem cells. Values are mean ± SEM. Significant effect of age, ^*^p < 0.1, ^**^p < 0.05. Scale bar, 200 μm.

Exponential growth rates of MuSCs isolated from young mice (young MuSCs) and aged MuSCs were determined to assess whether aged MuSCs were intrinsically impaired in their ability to proliferate. Young and aged MuSCs were cultured under standard culture conditions to expand the cell number and determine cell growth rates (Figure 1A). Individual growth curves of young and aged MuSCs over three passages were assessed (Figure 1B–1G). The P1 of young and aged MuSCs did not show difference in fold-change in cell number (Figure 1H). During subsequent passages (P2, or P3), no significant difference was found in fold-change in cell number over the initial cell number seeded, for young (2–5-fold) and aged (2–3-fold) MuSCs due to large coefficient of variation (Figure 1H). The average exponential growth rate of MuSCs from three passages (P1-P3) during the first two days of culture was determined. Since the young MuSCs reached confluence at day 2–3, the growth rate could not be determined at later time points. The growth rate of young MuSCs was 23% per day, while that of aged MuSCs was only 7% per day (Figure 1I).

To investigate whether the decrease in growth rate of aged MuSCs was accompanied by a decline in expression of myogenic and cell cycle genes, we determined gene expression of *Pax7*, *MyoD*, *Myog*, *Ccnd1*, *Cdk4*, and *Cdkn2a*. The percent difference between the means of young and aged MuSCs was quantified and is provided below. Compared to young MuSCs, *Pax7* gene expression was 44% lower and *Myog* expression was 198% higher in aged MuSCs, while no difference was observed in *MyoD* expression (Figure 1J). Assessment of cycle genes showed that the expression level of *Ccnd1* was 58% lower, and that of *Cdk4* was 40% lower in aged MuSCs than in young MuSCs, which agrees with the decreased aged MuSCs growth rate (Figure 1J). We then investigated whether the decrease in growth rate was accompanied by increased *Cdkn2a* expression in aged MuSCs. Our results showed 60% higher *Cdkn2a* gene expression (not significant) in aged MuSCs (Figure 1J). Gene expression of tissue inhibitor of metalloproteinase 1 (*Timp1*), which regulates matrix metalloproteinases [45], involved in MuSC migration was similar in young and aged MuSCs (Figure 1K). Moreover, gene expression of *c-fos*, which is involved in cell proliferation [46], was slightly (44%) but not significantly decreased in aged MuSCs (Figure 1L). We further analyzed gene expression of *IL-6*, which regulates proliferation, and found that aged MuSCs exhibited 77% lower *IL-6* expression (Figure 1M).

### Effects of age on MuSC morphology

**Figure 2:**
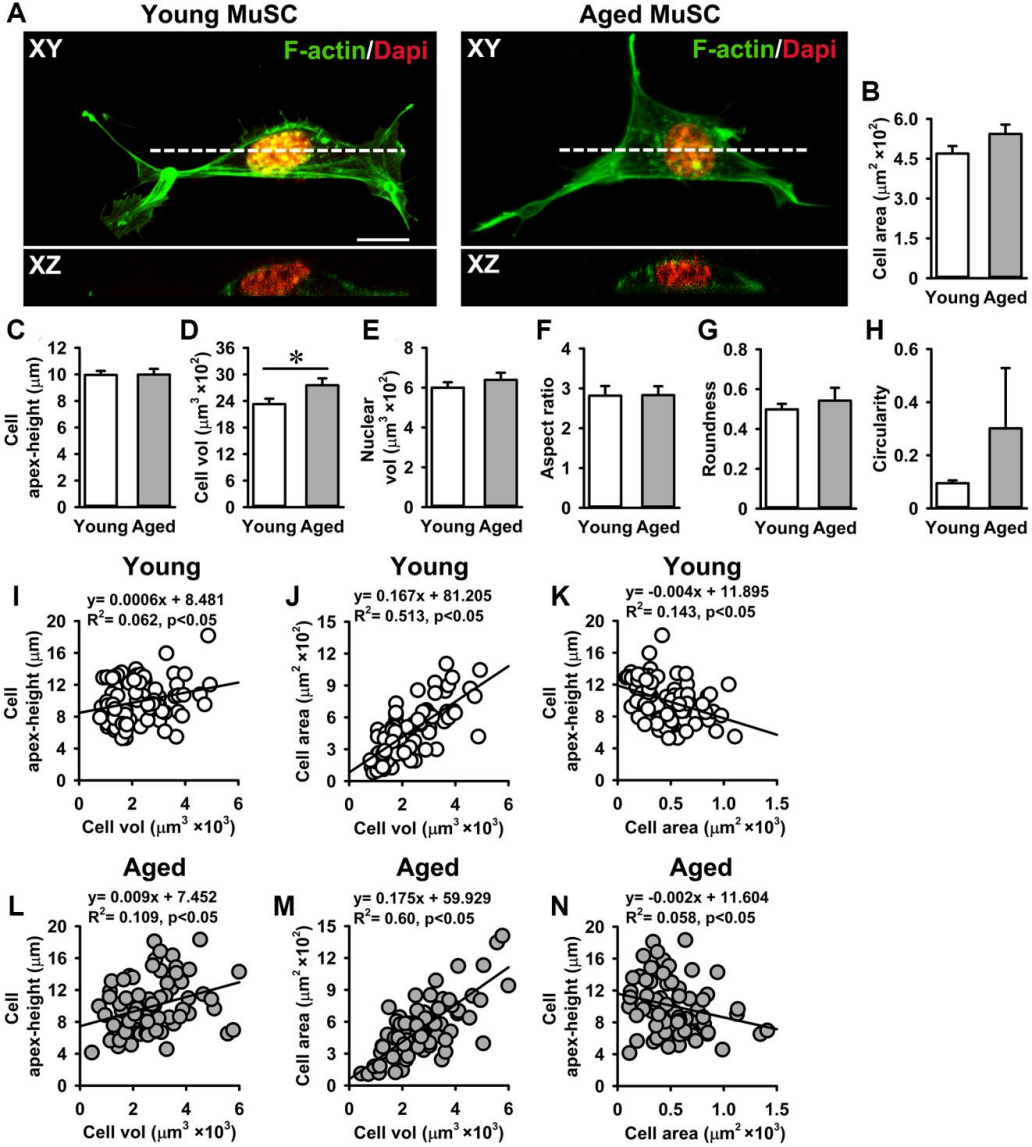
**Aged MuSCs exhibited a larger cell volume than young MuSCs.** (**A**) Top view (XY) and cross-sectional images (XZ, white dotted line) of young and aged MuSCs cultured for 3 days and stained for F-actin filaments (green) and nuclei (red) to quantify cell morphometry. (**B**, **C**) Cell spreading area and cell apex-height of MuSCs on matrigel coated glass substrate. (**D**, **E**) Aged MuSCs exhibited 18% larger cell volume than young MuSC whereas the nuclear volume did not differ. (**F**–**H**) Cell shape descriptors were not different between young and aged MuSCs. (**I**–**N**) Correlation between cell apex-height, cell volume and cell area of young and aged MuSCs. Abbreviation: MuSCs: muscle stem cells. Values are mean ± SEM. *n* = 75 cells (from 3 mice per age group). ^*^Significant effect of age, *p* < 0.05. Scale bar; 10 μm.

Cell volume is a tightly regulated process under a given growth condition. Cells with a large volume exhibit reduced proliferation and a senescent phenotype [47, 48]. This is in part attributed to an inefficient expression of cell cycle regulators [47]. Here we investigated whether the reduced growth rate of aged MuSCs was accompanied by a change in cell morphology. Confocal images revealed that cell adhesion area and apex-height did not differ between young and aged MuSCs (Figure 2A–2C). Next, we determined the cell and nuclear volume of MuSCs, and found that aged MuSCs exhibited 18% larger cell volume than young MuSCs, while no difference in nuclear volume was observed (Figure 2D, 2E). Other morphological properties, i.e. cell aspect ratio, roundness, and circularity were not different between young and aged MuSCs (Figure 2F–2H). On the other hand, the circularity of aged MuSCs had a large coefficient of variation of 652%, while for young MuSCs this value was only 97% (Figure 2H).

We tested whether MuSCs with a larger volume were more spread (i.e. flat) or round-shaped than MuSCs with a smaller volume by plotting the relation between cell apex-height, cell volume, and cell area. A slight positive relation between cell apex-height and cell volume was shown by both young (R^2^ = 0.06) and aged MuSCs (R^2^ = 0.10; Figure 2I, 2L). A strong positive correlation between cell area and cell volume was shown by both young (R^2^ = 0.51) and aged MuSCs (R^2^ = 0.60), indicating that MuSCs with a large cell volume spread more than MuSCs with a small volume *in vitro* (Figure 2J, 2M). In contrast, the relation between MuSC apex-height and cell area was negative in both young (R^2^ = 0.14) and aged MuSCs (R^2^ = 0.05; Figure 2K, 2N).

### Effect of mechanical loading on NO production and adhesion of MuSCs

**Figure 3:**
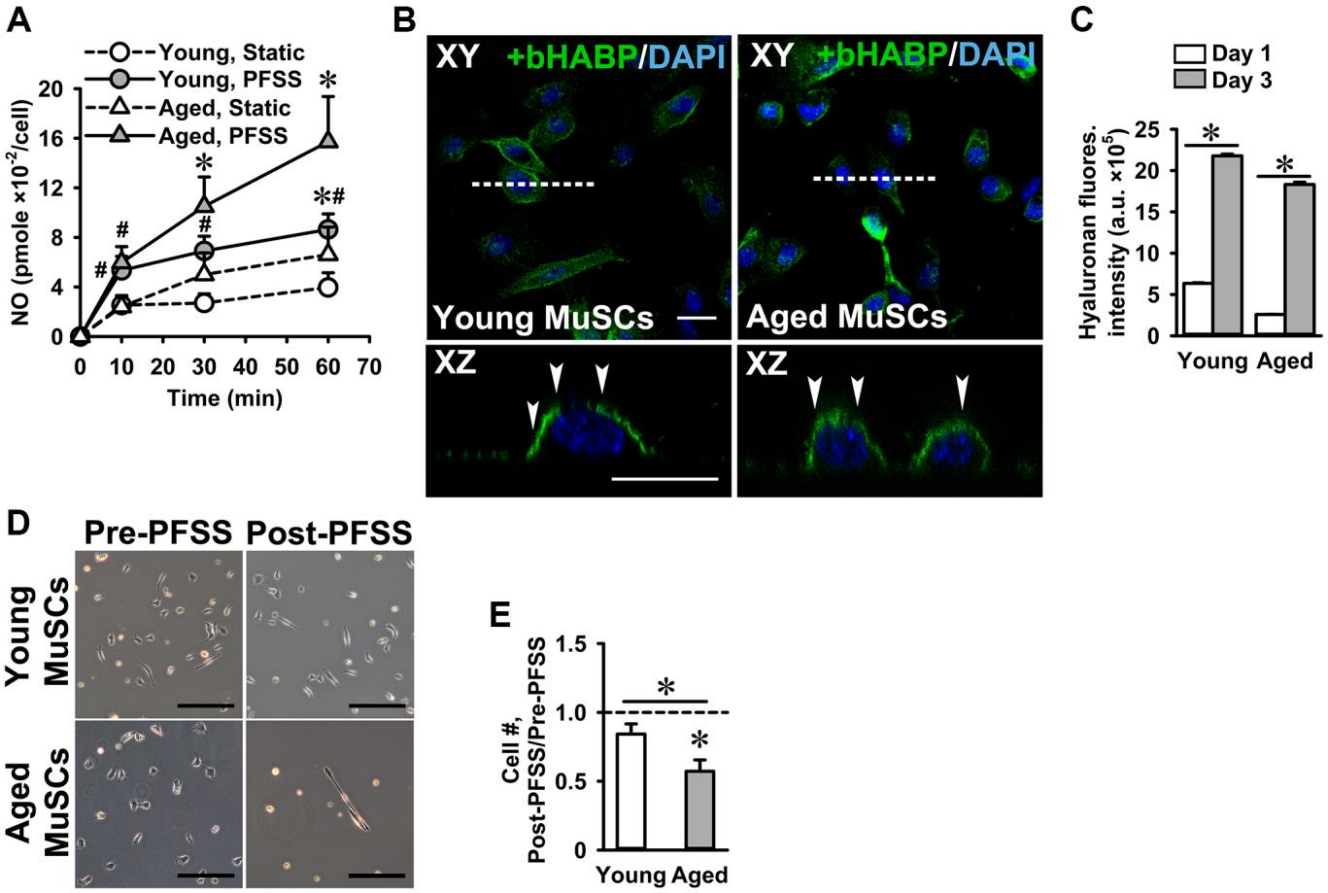
**PFSS-induced NO production and MuSC detachment.** (**A**) PFSS induced NO production in young and aged MuSCs. ^*^Significant effect of PFSS, ^#^Significantly different from static control, *p* < 0.05. Young MuSCs, *n* = 16–20 (from 4 young mice). Aged MuSCs, *n* = 14–17 (from 3 aged mice). (**B**) MuSCs stained for hyaluronic acid (glycocalyx component, green) and nuclei (blue). Top view (XY) and cross-sectional images (XZ, white dotted line) show that young and aged MuSCs expressed glycocalyx (white arrows). (**C**) Glycocalyx expression in young and aged MuSCs at day one and day three of culture. (**D**) Micrographs of MuSCs pre and post-PFSS treatment showed a decline in the number of aged MuSCs. Scale bar; 200 μm. (**E**) During 1 h PFSS (4.13 Pa/s), 43% of aged MuSCs detached from matrigel coated glass slides. Young MuSCs, *n* = 9 (from 3 young mice). Aged MuSCs, *n* = 12 (from 3 aged mice). Values are mean ± SEM. ^*^p < 0.05. Abbreviations: MuSCs: muscle stem cells; PFSS: pulsating fluid shear stress. Fluores. intensity, fluorescence intensity. Scale bar; 20 μm.

We have shown previously that C2C12 myotubes and osteocytes respond to pulsating fluid shear stress (PFSS)-induced mechanical loading by increased NO production [20, 49]. Here we investigated whether MuSCs also respond to mechanical loading by PFSS, and whether there is a difference in the response of young and aged MuSCs. PFSS stimulated NO production in young and aged MuSCs, while aged MuSCs showed a slightly higher (not significant) response compared to young MuSCs (Figure 3A). Ten minutes of PFSS increased NO production in young MuSCs by 2.1-fold and in aged MuSCs by 2.4-fold, after which the NO production did not further increase during 1 h PFSS treatment (Figure 3A). Earlier we have shown that NO production in C2C12 myotubes requires an intact glycocalyx [20]. Here we questioned whether primary MuSCs also have a glycocalyx, and whether this glycocalyx changes with age. We showed that both young and aged MuSCs expressed a glycocalyx (Figure 3B). Quantification of hyaluronic acid fluorescence intensity (a glycocalyx component) showed that glycocalyx expression increased by 3.5-fold in young MuSCs, and by 7.0-fold in aged MuSCs after three days of culture (Figure 3C). No significant difference was found in glycocalyx expression between young and aged MuSCs (Figure 3C). As cell shape and strength of attachment are related, i.e. well adhered and spread cells are likely to be more firmly attached than spherical cells [50], we also determined the strength of cell attachment by measuring the number of cells that detached from the substrate as a result of exposure to a shear force treatment. Fifteen percent of the young MuSCs detached during 1 h PFSS application, whereas 43% aged MuSCs were removed by PFSS (Figure 3D, 3E).

### Reduced adhesion of aged MuSCs is accompanied by lower focal adhesion number

**Figure 4:**
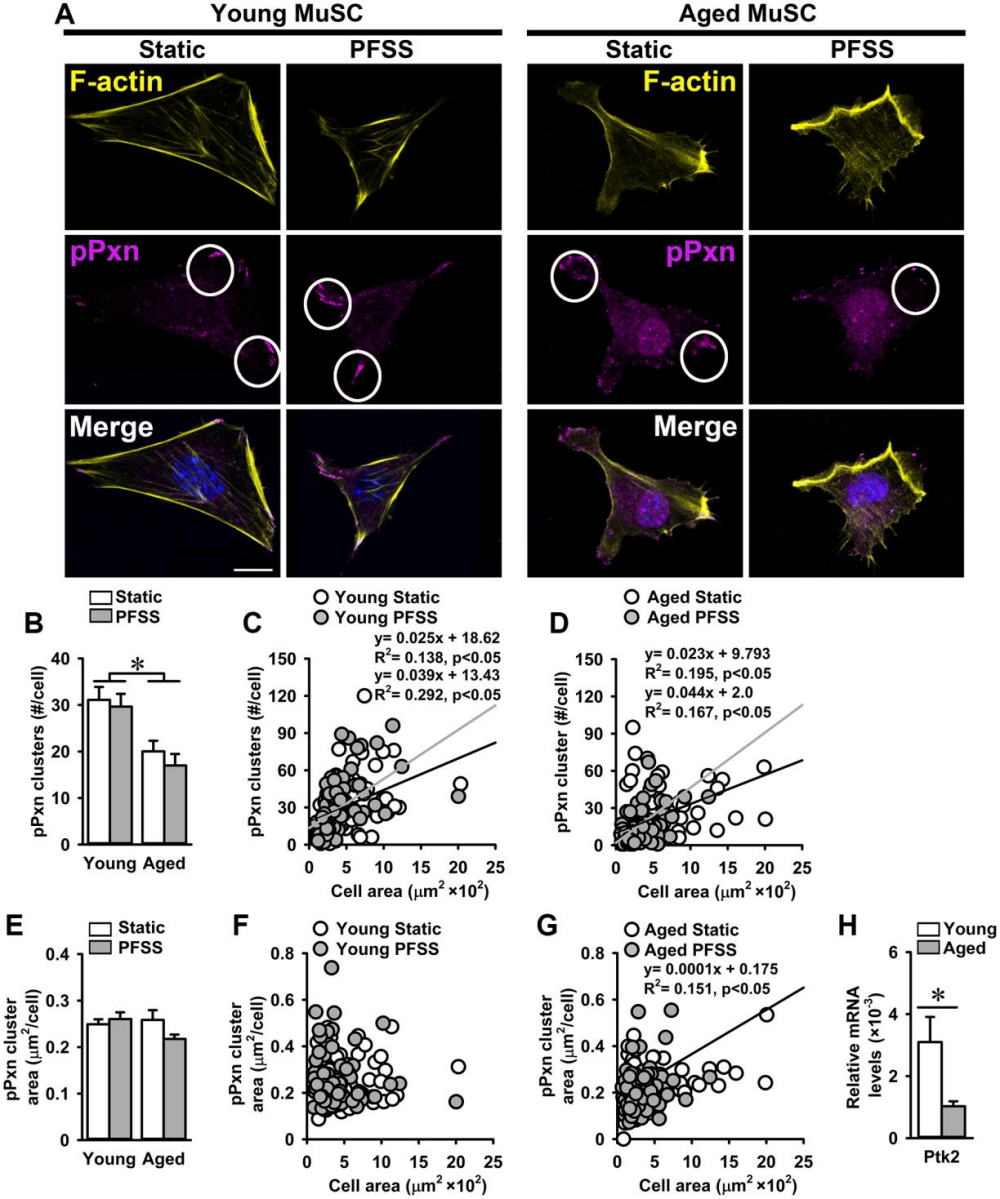
**Aging is associated with a decline in the number of focal adhesions in MuSCs.** (**A**) Young and aged MuSCs stained for phospho-paxillin (pPXN; magenta, white circles), F-actin filaments (yellow), and nuclei (blue) after 30 min of static and PFSS treatment. (**B**) Aged MuSCs illustrated lower number of pPXN clusters than young MuSCs and PFSS treatment did not affect the number of pPXN clusters. (**C**, **D**) Correlation between pPXN cluster number and cell attachment area in young and aged MuSCs. (**E**) pPXN cluster area in young and aged MuSCs after static and PFSS treatment. (**F**, **G**) Correlation between pPXN cluster area and cell attachment area in young and aged MuSCs. Young MuSCs, *n* = 63–74 (from 3 young mice). Aged MuSCs, *n* = 93–95 (from 3 aged mice). (**H**) Aged MuSCs exhibited lower gene expression of *Ptk2*. Young MuSCs, *n* = 11 (from 4 young mice). Aged MuSCs, *n* = 9 (from 3 aged mice). Abbreviations: MuSCs: muscle stem cells; PFSS: pulsating fluid shear stress. Values are mean ± SEM. ^*^Significant effect of age, *p* < 0.05. Scale bar: 10 μm.

Since a large number of aged MuSCs detached when exposed to PFSS, we investigated whether this was due to altered focal adhesion number and/or size, and whether MuSCs change focal adhesion number and/or size when subjected to mechanical loading. Therefore, young and aged MuSCs were stained for pPXN, and the number and size of focal adhesions was determined in PFSS-treated and untreated control cells. As expected, the number of pPXN clusters (focal adhesions) in aged MuSCs was 39% lower compared to that in young cells, while PFSS-treatment did not affect pPXN cluster number in young and aged MuSCs (Figure 4A, 4B). Furthermore, we assessed whether the number of pPXN clusters was related to cell attachment area. A positive relation between the number of pPXN clusters and cell area was shown for both young and aged MuSCs (young MuSCs, static: R^2^ = 0.13; PFSS: R^2^ = 0.29; (Figure 4C); aged MuSCs, static: R^2^ = 0.19; PFSS: R^2^ = 0.16; (Figure 4D)).

To determine whether the aging-related decline in the number of pPXN clusters was accompanied by a reduction in pPXN cluster size in MuSCs, the average pPXN cluster area per cell was determined. We also assessed whether PFSS-treatment affected the pPXN cluster size. Control and PFSS-treated young and aged MuSCs showed similar values for mean pPXN cluster area per cell (Figure 4E). Static control and PFSS-treated young MuSCs showed no relation between pPXN cluster area and cell attachment area (Figure 4F), while static aged MuSCs exhibited a positive relation between pPXN cluster area and cell area (R^2^ = 0.15; Figure 4G). Moreover, gene expression of *Ptk2* was also 68% lower in aged MuSCs, which coincided with a decreased number of focal adhesions (Figure 4H).

### Decreased integrin-α7 expression in aged MuSCs

**Figure 5:**
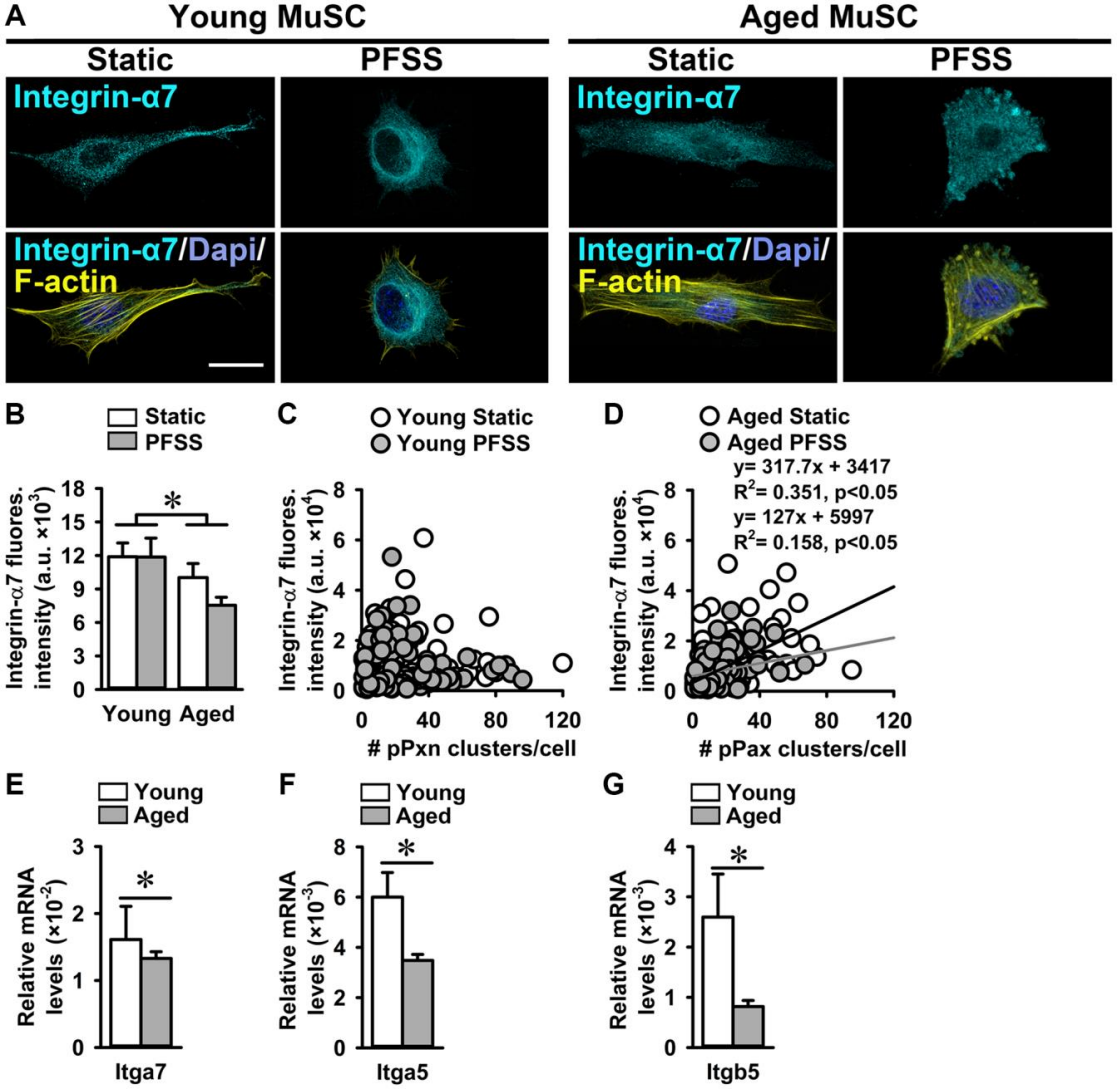
**Aged MuSCs showed reduced Integrin-α7 expression.** (**A**) Immunofluorescent images of young and aged MuSCs stained for Integrin-α7 (ITGA; cyan), F-actin filaments (yellow), and nuclei (blue) after 30 min of static and PFSS treatment. (**B**) ITGA7 fluorescent intensity was lower in static and PFSS-treated aged MuSCs compared to young cells. (**C**, **D**) Correlation between ITGA7 fluorescent intensity and number of pPXN clusters in young and aged MuSCs after static and PFSS. Young MuSCs, *n* = 63–74 cells (from 3 young mice). Aged MuSCs, *n* = 93–95 cells (from 3 aged mice). (**E**–**G**) Aged MuSCs exhibited lower *Itga7*, *Itga5*, and *Itgb5* gene expression than young MuSCs. Abbreviations: MuSCs: muscle stem cells; PFSS: pulsating fluid shear stress. Fluores. intensity, fluorescence intensity. Young MuSCs, *n* = 11 (from 4 young mice). Aged MuSCs, *n* = 9 (from 3 aged mice). Values are mean ± SEM. ^*^Significant effect of age, *p* < 0.05. Scale bar; 10 μm.

Integrins are transmembrane protein receptors that connect MuSCs to the ECM components and are part of focal adhesions [51]. Since we showed that aged MuSCs were less firmly attached to the substrate, we investigated whether ITGA7 levels are also reduced in aged MuSCs. MuSCs were subjected to 30 min of PFSS to determine whether mechanical loading induced integrin ITGA7 clustering. Confocal images of static control and PFSS-treated MuSCs, stained for ITGA7, revealed 17% lower ITGA7 expression in aged compared to young MuSCs as determined by the quantification of fluorescence intensity (Figure 5A, 5B). Furthermore, PFSS did not induce ITGA7 clustering within 30 min of PFSS (Figure 5A).

A possible relationship between ITGA7 fluorescence intensity and the number of pPXN clusters was assessed in young and aged static control and PFSS-treated MuSCs. We did not observe a relation between ITGA7 fluorescence intensity and the number of pPXN clusters in young MuSCs (Figure 5C). Interestingly, a group of young MuSCs showed a very high ITGA7 expression and low pPXN cluster number, while the other group had a very low ITGA7 expression and high pPXN cluster number (Figure 5C). In contrast, aged MuSCs showed a significant correlation between ITGA7 expression and pPXN cluster number in static control (R^2^ = 0.35) and PFSS-treated cultures (R^2^ = 0.35; Figure 5D). In line with the decreased ITGA7 fluorescent levels, *Itga7* gene expression was 17% lower in aged MuSCs compared to young MuSCs (Figure 5E). We quantified *Itga5* and *Itgb5* gene expression in young and aged MuSCs, and found decreased *Itga5* (42%) and *Itgb5* (68%) gene expression in aged MuSCs (Figure 5F, 5G).

### Increased YAP nuclearization in aged MuSCs

The reduced growth rate of aged MuSCs and their detachment due to PFSS-treatment, together with the lower number of focal adhesions and decreased ITGA7 levels compared to young MuSCs, suggest an impaired downstream signaling of ITGA7 receptors. Cells sense the mechanical properties of their niche via focal adhesions, which connect the outside environment to the cell cytoskeleton [52]. In mechanosensing, the hippo pathway and its effectors YAP and transcription regulator protein 1 (TAZ), which are transcription co-factors, play an important role by shuttling into the nucleus and induce upregulation of gene expression by binding to transcription factors. They determine MuSC fate and affect muscle regeneration [53]. YAP nuclearization is important in focal adhesion-related gene expression, and YAP knockout in a mesenchymal cell line disrupts focal adhesion formation and alters the actin cytoskeleton [38]. Here we investigated YAP nuclear localization in young and aged static control and PFSS-treated MuSCs.

**Figure 6:**
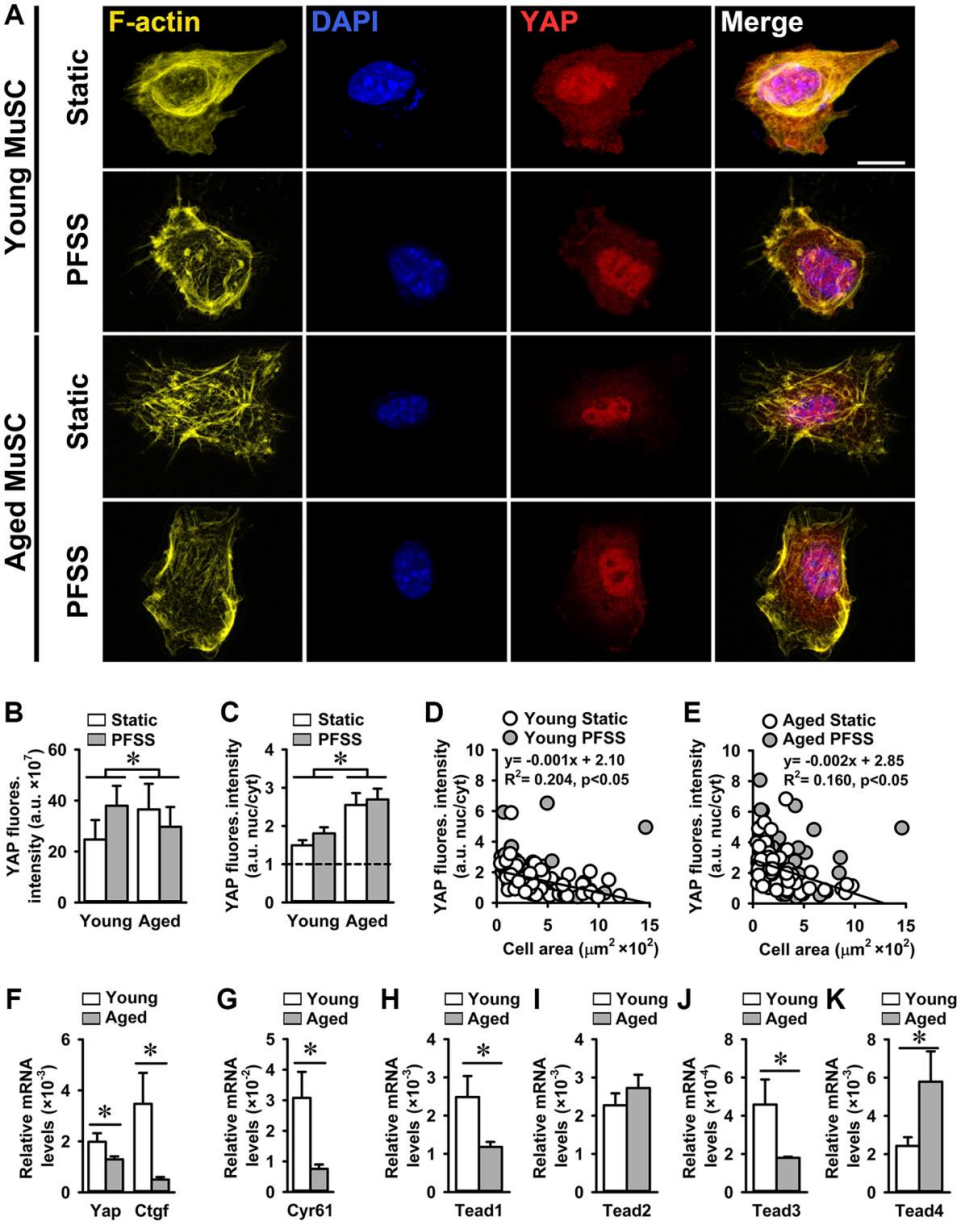
**Increased YAP nuclearization in aged MuSCs.** (**A**) Micrographs of young and aged MuSCs stained for YAP (red), F-actin filaments (yellow), and nuclei (blue) after 30 min of static culture or PFSS treatment. (**B**) Total YAP fluorescent intensity was higher in aged MuSCs. PFSS did not affect YAP fluorescent intensity in MuSCs. (**C**) YAP nuclear-to-cytoplasmic fluorescent intensity ratio revealed 59% higher YAP nuclear localization in aged MuSCs, and no effect of PFSS. (**D**, **E**) Negative correlation between YAP nuclear-to-cytoplasmic ratio and cell attachment area in young and aged MuSCs after static culture or PFSS treatment. Young MuSCs, *n* = 50–61 cells (from 3 young mice). Aged MuSCs, *n* = 55–56 cells (from 3 aged mice). (**F**, **G**) Gene expression *Yap* and its downstream targets *Ctgf* and *Cyr61* were decreased in aged MuSCs in comparison to young MuSCs. (**H**–**K**) Gene expression of *Tead1* and *Tead3* was decreased, *Tead2* was unchanged, and *Tead4* was increased in aged MuSCs. Young MuSCs, *n* = 11 (from 4 young mice). Aged MuSCs, *n* = 9 (from 3 aged mice). Abbreviations: MuSCs: muscle stem cells; PFSS: pulsating fluid shear stress. Fluores. intensity, fluorescence intensity. Values are mean ± SEM. ^*^Significant effect of age, *p* < 0.05. Scale bar; 10 μm.

Confocal images of young and aged MuSCs stained for YAP, nuclei, and actin cytoskeleton, showed YAP nuclearization in static control and PFSS-treated cells (Figure 6A). Total YAP in aged static control and PFSS-treated MuSCs was 5.3% higher than in young MuSCs (Figure 6B). Young, but not aged MuSCs treated with PFSS showed an upward trend in YAP intensity compared to static controls (Figure 6B). We also determined YAP nuclear localization, and contrary to our expectations, aged MuSCs exhibited a 59% higher nuclear YAP fluorescence intensity than young MuSCs (Figure 6C). PFSS did not significantly change the YAP content within the nucleus of MuSCs, but in young MuSCs YAP nuclearization after PFSS treatment was slightly, but not significantly increased by 21% (Figure 6C).

YAP nuclearization in mesenchymal stem cells depends on cell attachment area irrespective of focal adhesions assembly [38]. Here we determined to what extent the cell attachment area determines YAP localization in MuSCs. In contrast to previous finding [38], we found that both young and aged MuSCs with a smaller attachment area exhibited higher YAP nuclearization (young MuSCs: R^2^ = 0.20; aged MuSCs: R^2^ = 0.16 (Figure 6D, 6E)). Aged MuSCs exhibited 35% lower *Yap* gene expression than young MuSCs (Figure 6F). Expression of YAP downstream targets *Ctgf* and *Cyr61* was decreased by 85% and 75%, respectively in aged MuSCs (Figure 6F, 6G). We also assessed whether declined YAP target gene expression in aged MuSCs was due to decreased *Tead* gene expression. We found that gene expression of *Tead1* and *Tead3* was decreased (52% and 61%, respectively), *Tead2* remained unchanged, and *Tead4* increased (138%; Figure 6H–6K).

### MuSCs mechanoresponsiveness to PFSS

**Figure 7:**
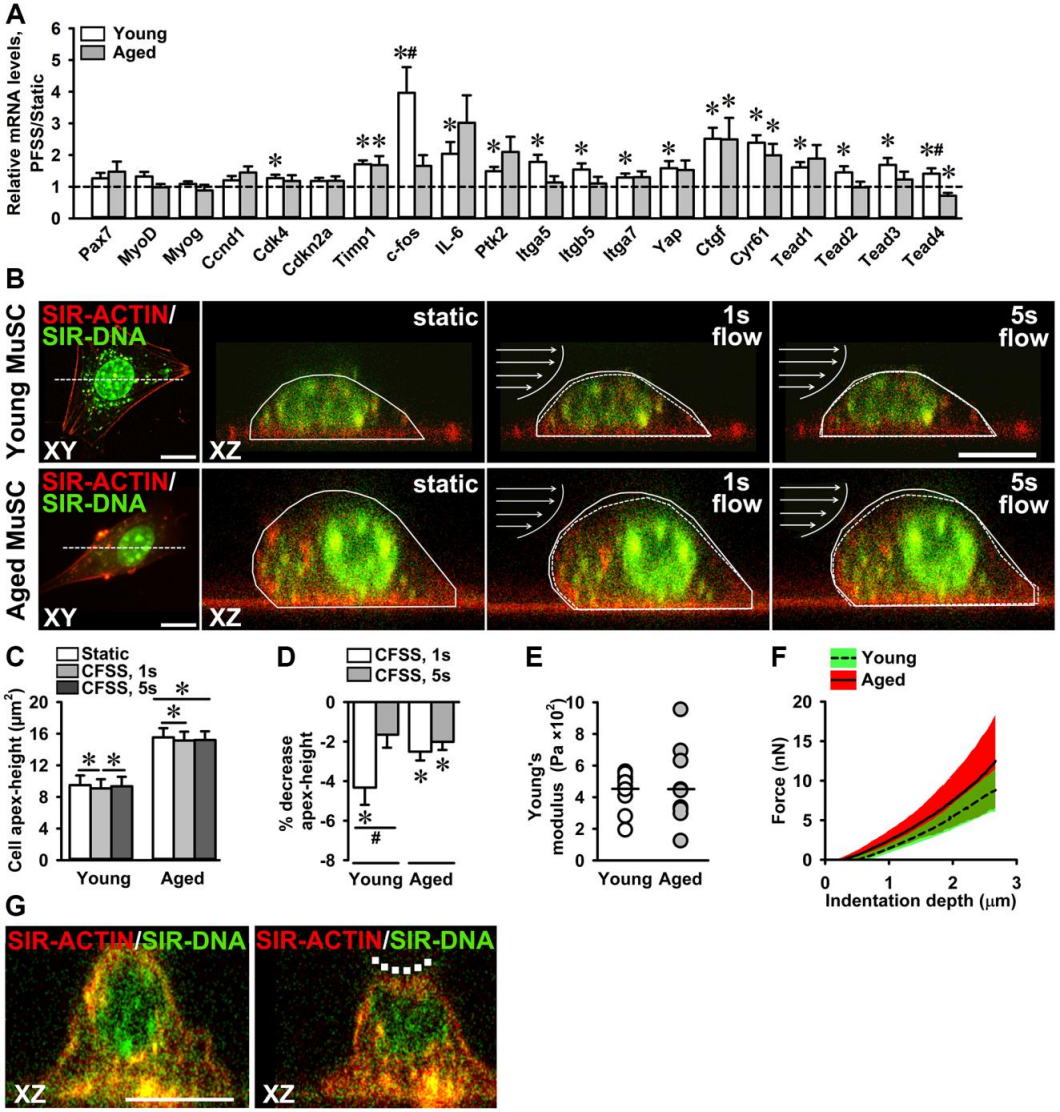
**Attenuated mechanosensitivity to PFSS in aged MuSCs.** (**A**) MuSC gene expression in response to PFSS, showing upregulation of *Cdk4*, *Timp1*, *c-fos*, *IL-6*, *Ptk2*, *Itga5*, *Itgb5*, *Itga7*, *Yap*, *Ctgf*, *Cyr61*, *Tead1*, *Tead2*, *Tead3*, and *Tead4*, in young MuSCs, and *Timp1*, *Ctgf*, and *Cyr61* in aged MuSCs. Young MuSCs, *n* = 10 (from 4 young mice). Aged MuSCs, *n* = 8 (from 3 aged mice). (**B**) Confocal top view (XY) and cross-sectional (XZ) live cell images of young and aged MuSCs stained for F-actin filaments (red), and nuclei (green), before (static) and during (1 s, 5 s) fluid shear stress treatment, illustrating the change in cell apex-height as a result of fluid shear stress. The change in cell apex-height is highlighted with solid and dotted lines in XZ images. Arrows indicate the direction of fluid flow (**C**, **D**) CFSS induced MuSC deformation and decreased the cell apex-height of young and aged MuSCs at the start of CFSS treatment (1 s). Aged MuSCs remained deformed after 5 s of treatment, whereas young MuSCs regained their initial cell apex-height. Young MuSCs, *n* = 13 (from 3 young mice). Aged MuSCs, *n* = 10 (from 3 aged mice). (**E**) Nano-indentation of MuSCs revealed a similar Young’s modulus (~450 Pa) of young and aged MuSCs. The coefficient of variation was large in aged MuSCs (61%) compared to young MuSCs (27%). (**F**) Force-indentation curves showing large variation in force required to indent aged MuSCs compared to young MuSCs. (**G**) Confocal cross-sectional micrographs of young MuSC stained for F-actin filaments (red) and nucleus (green) before and during nano-indentation, illustrating cell deformation (dotted line). Young and aged MuSCs, *n* = 10 cells (from 1 young mouse and 1 aged mouse). Abbreviations: MuSCs: muscle stem cells; PFSS: pulsating fluid shear stress; CFSS: constant fluid shear stress. Values are mean ± SEM. ^*^Significant effect of PFSS, *p* < 0.05. ^#^Significant difference between age groups, *p* < 0.05. Scale bar; 10 μm.

To investigate whether PFSS-treatment induced expression of genes related to proliferation, focal adhesion, and YAP signaling, and whether mechanoresponsiveness was compromised with age, gene expression in static control and PFSS-treated young and aged MuSCs was determined. PFSS upregulated gene expression of *Cdk4* (27%), *Timp1* (71%), *c-fos* (296%), *IL-6* (104%), *Ptk2* (49%), *Itga5* (78%), *Itgb5* (54%), *Itga7* (29%), *Yap* (58%), *Ctgf* (151%), *Cyr61* (139%), *Tead1* (60%), *Tead2* (45%), *Tead3* (69%), and *Tead4* (41%) in young MuSCs (Figure 7A). In aged MuSCs, PFSS only upregulated gene expression of *Timp1* (68%), *Ctgf* (149%), and *Cyr61* (99%; Figure 7A). *Tead4* gene expression was reduced by 29% after PFSS treatment in aged MuSCs (Figure 7A). The coefficient of variation for PFSS-induced gene expression was higher in aged MuSCs i.e. *Pax7* (61%), *Myog* (57%), *Cdk4* (43%), *Cdkn2a* (35%), *Timp1* (48%), *IL-6* (81%), *Ptk2* (65%), *Itgb5* (54%), *Itga7* (40%), *Yap* (56%), *Ctgf* (77%), *Cyr61* (51%), *Tead1* (64%), and *Tead3* (57%). Whereas young MuSCs exhibited a lower coefficient of variation for these genes i.e. *Pax7* (43%), *Myog* (24%), *Cdk4* (28%), *Cdkn2a* (26%), *Timp1* (22%), *IL-6* (55%), *Ptk2* (28%), *Itgb5* (39%), *Itga7* (31%), *Yap* (45%), *Ctgf* (44%), *Cyr61* (32%), *Tead1* (31%), and *Tead3* (39%). The response to loading has been linked to cell shape, as explored above, but also to cell stiffness [54, 55]. To explore whether aged MuSCs exhibited an altered cell stiffness, live cell imaging of MuSCs subjected to a constant fluid shear stress (CFSS) was performed and cell deformation was quantified. We showed that young and aged MuSCs deform in response to shear stress (Figure 7B). At the start of shear stress treatment (at 1 sec CFSS), cell apex-height was reduced in young MuSCs by 4.3%, and in aged MuSCs by 2.5%. After 5 sec CFSS application, young MuSCs almost regained their initial apex-height, but aged MuSCs remained deformed (Figure 7C, 7D). We then determined the Young’s moduli of MuSCs by indentation of MuSCs using a nano-indenter with spherical tip (diameter: 7 μm). Our results revealed a similar Young’s modulus (~450 Pa) of young and aged MuSCs, but aged MuSCs exhibited a substantially larger coefficient of variation (61%) compared to that of young MuSCs (27%; Figure 7E–7G). As evident from the force-indentation curves of MuSCs, large variation in the indentation force was observed for aged MuSCs (Figure 7F).

## DISCUSSION

Loss of MuSC number and function are considered limiting factors in skeletal muscle regeneration after injury in aged individuals, leading to sarcopenia [56]. Several studies suggest that niche conditions and systemic factors are critical in age-related declined MuSC function [9–14]. The current study aimed to elucidate whether aged MuSCs are intrinsically impaired in their growth rate, and to test whether aging alters MuSC adhesion and mechanosensitivity by modulation of ITGA7 expression, focal adhesion number, as well as the hippo signaling. Moreover, we aimed to determine whether aged MuSCs exhibit an altered expression of genes crucial for MuSC regenerative function, and whether MuSCs respond to mechanical loading by NO production and upregulation of genes related to cell cycle, focal adhesion, integrin, and the hippo pathway. This study shows that aged MuSCs exhibited a reduced growth rate despite high nuclear YAP levels and, increased cell volume. In addition, aged MuSCs showed decreased adhesion, reduced ITGA7, and focal adhesion number. Moreover, aged MuSCs had lower basal expression of genes essential for MuSC function, and exhibited altered mechanosensitivity when exposed to mechanical loads. These data suggest that aged MuSCs are intrinsically impaired and exhibit altered adhesion and mechanosensitivity which may contribute to the reduced growth rate of these cells.

### Impaired growth rate of aged MuSCs

Reduced proliferation might be a limiting factor in declined regenerative capacity of skeletal muscles with age [57]. In the current study, we show that despite a high YAP nuclear localization, aged MuSCs exhibited a significantly decreased growth rate *in vitro* compared to young MuSCs. Aging is associated with alterations in gene expression profiles of MuSCs [58]. Elevated cell cycle inhibitor CDKN2A (P16) is a cell senescent marker in aged cells, and its expression has been shown to be increased in aged MuSCs [59]. We showed that aged MuSCs exhibited lower gene expression of *Pax7*, *Ccnd1*, *Cdk4*, and higher *Myog* expression, whereas *Cdkn2a* expression was slightly but not significantly higher in aged MuSCs compared to young MuSCs. Contrary to previous findings that IL-6 serum levels are elevated with age [60], our results showed that *IL-6* gene expression was lower in aged MuSCs, suggesting that aged MuSCs were intrinsically altered in the signaling pathways governing proliferation and MuSC function. The following studies suggest that changes in cell-extrinsic factors affect MuSC proliferation and function. Increased ECM stiffness induces expression of pathogenic matricellular proteins by fibroblasts due to increased YAP/TAZ activity, which alters MuSC function [53]. Chakkalakal et al. [10] reported that the aged niche disrupts aged MuSC quiescence. Moreover, loss of fibronectin in the aged niche disrupts MuSC adhesion to the ECM, and integrin-mediated signaling [56]. Parabiosis studies emphasize the importance of systemic factors for MuSC function [14]. However, the declined MuSC function resulting from aging as found in this study cannot be explained by external factors alone, as they were cultured in same culture conditions outside the aged niche. Mitogen-activated kinases are elevated in aged MuSCs causing a defect in MuSC self-renewal, which cannot be reversed by transplantation into a young niche [59]. Reduced Notch signaling with age also alters a proper MuSC regenerative function [61]. Our study supports the notion that besides extracellular factors, several intrinsic changes in MuSCs lead to decreased MuSC growth rate and altered function. Below we discuss these intrinsic changes in aged MuSCs that may contribute to reduced MuSC growth rate and defective muscle regeneration.

### Increased cell volume of aged MuSCs

Cellular physical properties including area, apex-height, shape, nuclear volume, and cell volume are tightly regulated, and are influenced by various stimuli such as substrate stiffness, osmotic change, mechanical perturbations like shear stress, and biochemical signals [62, 63]. Changes in these cellular parameters can affect stem cell function and fate decisions [62]. Therefore, we investigated whether the reduced growth rate of aged MuSCs was also accompanied by alterations in the morphological properties of the cells. We found that the cell volume was increased in aged MuSCs compared to young MuSCs. This increase in cell volume has implications for the mechanical properties and mechanosensitivity of MuSCs. A decrease in cell volume by water efflux increases cell stiffness and macro-molecular density, thus altering the mechanical properties of a cell [62]. Moreover, alterations in cell volume affect protein folding and intracellular dynamics, including binding kinetics and signaling pathways which may influence MuSC differentiation [62, 64, 65]. Our findings that aged MuSCs exhibit increased nuclear YAP localization and increased cell volume are in line with observations by others that the cell volume is positively correlated to the cell apical tension and nuclear levels of YAP/TAZ [42].

The morphology of a cell is in part determined by the interaction of the cell with its environment and the mechanical properties of the cytoskeleton [66]. It is likely that cells of varying morphology and volume have variable mechanical properties, and that the response of cells with varying morphology to mechanical force differs as well [67, 68]. We determined whether the increased aged MuSC volume was accompanied by a reduction in focal adhesion number. Our results suggest that the increase in aged MuSC volume may be, at least in part, the result of changed focal adhesion assembly and/or cytoskeleton. This may affect the mechanical properties and mechanosensitivity to the acting physical forces within the niche of aged MuSCs.

### Mechanical load-induced NO production, and adhesion of MuSCs

NO is an important signaling molecule involved in the activation of MuSCs during muscle regeneration by activating matrix metalloproteinases and releasing hepatocyte growth factor from the ECM [21, 69]. Previously we have shown that an intact glycocalyx is required for PFSS-induced NO production in C2C12 myotubes, and that removal of the glycocalyx ablates the effect of PFSS on NO production [20]. Since NO levels in static young and aged MuSC cultures were similar, the decreased growth rate of aged MuSCs could not be explained by the differences in NO production. We found that young and aged MuSCs similarly responded to PFSS with enhanced NO production. Whether inhibition of NO would result in differences in young and aged MuSC activity is currently unknown.

Decreased adhesion of hematopoietic stem cells with their cell niche negatively affects stem cell number and function [70]. In the current study, aged MuSCs were less firmly attached to the matrigel-coated glass substrate compared to young MuSCs, while a high number of MuSCs detached due to PFSS application. The decreased number of focal adhesions and lower ITGA7 expression in aged MuSCs may explain the diminished cell adhesion to the matrigel-coated glass substrate. Moreover, the decreased *Ptk2* gene expression in aged MuSCs agrees with the decreased cell adhesion. Cell-matrix adhesion plays a role in the proliferation and morphology of pluripotent stem cells [71]. We conclude that the reduced growth rate of aged MuSCs is, at least in part, due to decreased cell adhesion.

### Implications of reduced focal adhesion number and integrin-α7 expression in aged MuSCs

The cytoplasmic domain of integrins is attached to a number of adapter proteins, i.e. PTK2 and paxillin that are involved in actin cytoskeletal organization, adhesion, migration, proliferation, regulating cell shape, and transmission of biochemical and mechanical signals into the cells [72–74]. Integrin attachment to the ECM signals paxillin recruitment for focal adhesion formation, followed by PTK2-mediated paxillin phosphorylation [31]. Fibroblasts of aged mice exhibit altered focal adhesion formation and cytoskeleton, accompanied by low mobility and proliferation [75]. Loss of PTK2 activity impairs focal adhesion turnover [73]. In line with these studies, we show in the current study a declined *Ptk2* gene expression and a decreased number of pPXN clusters in aged MuSCs suggesting that the reduced growth rate of aged MuSCs and loss of mechanosensitivity is, at least in part, due to a diminished focal adhesion formation and/or turnover.

Loss of fibronectin from the aged MuSC niche causes a decline in β1-integrin-mediated signaling via PTK2, and has been implicated as one of the causes of aged MuSC loss of function [56]. Moreover, β1-integrin plays an essential role in MuSC proliferation [24]. We showed that both *Itga7* gene expression and ITGA7 protein expression were decreased in aged MuSCs. An explanation for the lower ITGA7 protein expression in aged MuSCs could be the decreased *Itga7* gene expression levels. Although cytoplasmic and membrane ITGA7 was detected, it is unknown whether protein translocation from cytoplasm to cell membrane was changed. *Itga5* and *Itgb5* gene expression were also declined in aged MuSCs. Β1-integrin-knockout (*Itgb1*−/−) mice exhibit impaired adhesion and PTK2 signaling similar to aged MuSCs [56]. This suggests that decreased ITGA7 expression and focal adhesion formation are involved in altered MuSC function with age, possibly due to niche changes leading to intrinsic changes in MuSCs. ITGA7 and pPXN present potential therapeutic targets to improve aged MuSC function.

### High YAP nuclear localization in aged MuSCs

Hippo signaling pathway and its core effector YAP regulate tissue growth, stem cell self-renewal, and expansion [35, 36]. Therefore, YAP expression has been a target to enhance proliferation and tissue growth. Inhibition of YAP-TEAD interaction reduces TEAD target gene expression, proliferation, and cell migration [36]. On the other hand, constitutive YAP expression in skeletal myofibers induces muscle atrophy [76]. Aged myofibroblasts exhibit elevated YAP levels and express ECM proteins that disrupt MuSC function [53], similar to YAP expression in aged MuSCs as shown in this study. We showed that despite a higher nuclear localization of YAP in aged MuSCs compared to young MuSCs, growth rate, focal adhesion formation, and MuSC function were altered in aged MuSCs. In line with these results, the Wnt/β-catenin signaling-mediated YAP upregulation induces a sarcopenia phenotype via the (pro)renin receptor in a sarcopenia mouse model [77]. Since YAP is also regulated via cell-cell contact inhibition, actin cytoskeleton, and myosin contractility [78], the differential effects of YAP upregulation in these studies may have different mechanistic causes.

YAP nuclear-to-cytoplasmic ratio is the key determinant in its activity and also a target of regulation by the Hippo signaling pathway [79]. Hippo pathway kinases phosphorylate and retain YAP to cell cytoplasm [79]. However, YAP activity is also regulated via other diverse mechanisms i.e., mechanical forces and ECM stiffness [32–34, 37], cell-cell contact, cytoskeletal integrity [78], and YAP protein modifications [80, 81]. Aging is associated with increased fibrosis and ECM stiffness of MuSC niche [12, 25]. Stiff substrates induce high YAP nuclear localization [32–34]. The high nuclear localization in aged MuSCs shown in this study can likely be due to the stiff aged MuSC niche and the mechanical memory of these cells to their *in vivo* niche. Mesenchymal stem cells maintained on stiff substrate for ten days, retain nuclear YAP localization even after removal of these cells from stiff substrate [82]. Moreover, in *Saccharomyces cerevisiae* and *Caenorhabditis elegans* aging-associated dysfunction of nuclear pore complex has been reported [83]. This can likely lead to altered nuclear shuttling of transcription factors and increased nuclear localization [84]. Aged mice myofibers also show increased YAP activation and altered expression of nuclear pore complex proteins i.e. Nup107 and Nup93 [84]. However, in aged MuSCs such alterations in nuclear pore complex has not been reported yet. Biochemical changes within the aged MuSC niche can also induce altered YAP signaling via protein modifications [80, 81], or via changes in the MuSC metabolic pathways i.e. glucose and lipid metabolism [85, 86]. Moreover, YAP acts as a mechanotransducer of physical loads and it has been proposed that mechanical cues can activate YAP independent of the Hippo pathway [87]. Aging-associated alteration in mechanosensitivity and MuSC stiffness can likely contribute to the altered YAP signaling.

YAP activation enhances TEAD target gene expression, i.e. *Ctgf* and *Cyr61*, and promotes cell proliferation and migration [35, 41]. YAP hyper-activation has been shown to induce cellular senescence in human ovarian cells via the YAP-LATS2 feedback loop [88]. Here we showed that *Yap* gene expression was decreased, and YAP nuclear localization increased in aged MuSCs compared to young MuSCs. However, the expression of *Ctgf* and *Cyr61*, downstream targets of YAP were significantly reduced in aged MuSCs. Decreased *Tead1* and *Tead3* gene expression was observed in aged MuSCs, which was in line with decreased expression of *Yap* and its target genes. We found increased *Tead4* gene expression, which might be due to increased *Myog* gene expression in aged MuSCs. MYOG protein directly interacts with the *Tead4* promoter and upregulates *Tead4* gene expression during C2C12 myoblast differentiation [89]. TEAD4 protein interacts with the *MyoD* promoter and plays an essential role during C2C12 myoblast differentiation [90]. Silencing of *Tead1* in combination with *Tead4*, but not *Tead1* or *Tead4* gene expression, impairs C2C12 myoblast differentiation [91]. Since YAP signaling is involved in MuSC proliferation, our results on *Tead* and *YAP* gene expression suggest that the declined aged MuSC growth rate could be due to altered YAP signaling. Moreover, the increased *Tead4* and *Myog* gene expression suggests that aged MuSCs upregulated differentiation-related gene expression.

### Altered mechanosensitivity of aged MuSCs

MuSCs are subjected to tensile and shear deformations within their niche during myofiber stretch-shortening [18]. These mechanical loads acting on MuSCs are suggested to play an essential role in MuSC function [92, 93]. We showed that young MuSCs subjected to shear forces upregulated expression of genes involved in regulation of adhesion, cell cycle, and MuSC function. Aged MuSCs were less sensitive to shear forces and showed upregulation of less genes, suggesting that the decreased mechanosensitivity was due to decreased integrin protein expression, i.e. ITGA7, ITGA5, and ITGB5, and focal adhesion number. In aged MuSCs, gene expression of downstream targets of YAP, i.e. *Ctgf* and *Cyr61*, was increased by mechanical loading, whereas *Tead4* expression was decreased. Moreover, in aged MuSCs, PFSS-induced gene expression exhibited a high coefficient of variation compared to young MuSCs. This suggests that aged MuSCs exhibit a heterogenous mechanosensitivity which can likely be due to a heterogenous expression of mechanosensitive proteins (i.e., integrins and paxillin). Whether long-term mechanical loading of aged MuSCs reverts altered YAP signaling and induces MuSC proliferation by downregulation of differentiation-associated genes, i.e., *Tead4*, *Myog*, is unknown. We observed a difference in mechanoresponsiveness between young and aged MuSCs under similar, well-defined culture conditions. This suggests that the decreased mechanoresponsiveness of aged MuSCs *in vitro* might be due to aging-associated cell-intrinsic alterations. Moreover, there is evidence suggesting that the decline in MuSC functionality with increasing age is driven by intrinsic changes (e.g. upregulation of developmental pathways). It is also known that MuSC functionality declines *in vivo* as a result of changes in the stem cell niche [10, 56]. However, our MuSCs were isolated, i.e. outside their *in vivo* niche under well-defined conditions in the presence of medium supplements in which young and aged MuSCs function normally. We cannot exclude that our MuSCs memorize their *in vivo* niche, but this also implies that the aged niche conditions altered the MuSCs intrinsically. *In vivo* evidence supporting our claim of reduced mechanoresponsiveness in aged MuSCs would be important. Future studies should be dedicated to *in vivo* validation.

The mechanical properties of the cytoskeleton are crucial for cell adhesion, division, mobility, structure, and mechanotransduction [94]. We also showed that shear forces caused deformation of young and aged MuSCs. Interestingly, aged MuSCs remained deformed under shear forces, while young MuSCs regained their initial cell apex-height. Moreover, aged MuSCs showed higher variation in cell stiffness as shown by force-induced cell indentation which suggests that aged MuSCs have an altered cytoskeleton. Taken together, these results indicate that aged MuSCs are less mechanoresponsive to PFSS, suggesting an altered mechanosensitivity in aged cells. Analysis of the F-actin cytoskeleton and stiffness of MuSCs during aging will shed more light into the mechanobiology of the aged MuSC cytoskeleton. Moreover, MuSCs consist of different sub-populations [95, 96]. MuSCs within slow oxidative muscle differ in morphology compared to cells within fast glycolytic muscle [18]. Whether these sub-populations of MuSCs are differently affected by aging and exhibit differential expression of mechanosensitive molecules and hence different mechanoresponsiveness is unknown. Further studies are required to provide more insight into the possible effect of aging on different MuSC sub-populations.

## CONCLUSIONS

In this study we showed that aged MuSCs were intrinsically impaired in their growth rate. The mechanical link between the outer environment and cell interior was substantially affected by age due to decreased ITGA7 expression and diminished focal adhesion formation, which coincided with an increased cell volume, decreased MuSC adhesion, and altered mechanosensitivity of the cells to mechanical loads. Moreover, YAP signaling was changed in aged MuSCs, and the expression of several genes including cell cycle genes was decreased. As an implication, a possible therapeutic option could be restoration ITGA7 and focal adhesion number in aged MuSCs, which may help to restore MuSCs adhesion to their niche as well as growth rate of these cells.

## MATERIALS AND METHODS

### Primary MuSC isolation and fluorescence-activated cell sorting

Animal procedures were conducted according to the European Community guidelines. Experimental animal protocols were performed in accordance with the guidelines of the French Veterinary Department and approved by the Sorbonne Université Ethical Committee for Animal Experimentation.

Primary MuSCs were isolated from young (2 months; *n* = 4) and aged (22 months; *n* = 3) male mouse (C57BL/6J) hindlimb muscles (Hamstring muscle group, Quadriceps, Tibialis, Extensor digitorum longus, Gastrocnemius, Soleus, Gluteus) after enzymatic digestion followed by Fluorescence-activated cell sorting (FACS) purification. The isolation of young and aged MuSCs was performed as described earlier [97, 98]. MuSCs were isolated as CD31^−^, CD45^−^, Sca1^−^, α7 integrin^+^, and CD106^+^. It has been shown that these surface markers, or lack thereof, are efficient in isolating young and aged MuSCs [98]. This suggests that our isolated MuSCs adequately represent young and aged MuSCs. Briefly, after dissection muscles were first digested with collagenase II (1000 U/ml; Worthington Biochemical Corporation, Lakewood, NJ, USA) in Ham’s F10 containing 10% horse serum, washed and then further digested with collagenase II (1000 U/ml) and dispase (11 U/ml) for 30 min. After digestion cells were washed in Ham’s F10 containing 10% horse serum, passed 10 times through a 20-gauge needle syringe and then filtered with a 35-mm cell strainer (Falcon^®^, Corning, NY, USA). Cells were then stained with the following antibodies: rat CD31-eFluor450 (1/500; eBiosciences™, Thermo-Fisher Scientific, San Diego, CA, USA), rat CD45-eFluor450 (1/500; eBiosciences™, Thermo-Fisher Scientific), rat Ly6A-FITC (SCA1) (1/500; eBiosciences™, Thermo-Fisher Scientific), rat CD106-PE (1/200; eBiosciences™, Thermo-Fisher Scientific) and rat α7 integrin-APC (1/1000; AbLab, Vancouver, Canada) and sorted using a FACS Aria II (BD Biosciences, San Jose, CA, USA). Satellite cells were isolated as CD31^−^, CD45^−^, Sca1^−^, α7 integrin^+^, CD106^+^.

### MuSC culture

MuSCs were expanded on matrigel (Corning, Bedford, MA, USA)-coated culture flasks with growth medium consisting of Ham’s F-10 Nutrient Mix (Gibco, Paisley, UK) supplemented with 20% fetal bovine serum (FBS; Gibco), 10 μg/ml penicillin (Sigma-Aldrich, St. Louis, MO, USA), 10 μg/ml streptomycin (Sigma-Aldrich), and 2.5 ng/μl of recombinant human fibroblast growth factor (R&D systems, Minneapolis, MN, USA), and cultured at 37°C in a humidified atmosphere of 5% CO_2_ in air. Upon 70% confluence, cells were harvested using 0.1% trypsin and 0.1% EDTA (Gibco) in PBS.

### MuSC proliferation and growth rate

For cell counting, an adopted non-invasive imaging method was used as described [99]. Briefly, every 24 h pictures of cell cultures were taken at predetermined positions within the culture flasks, using a Zeiss Axiovert microscope with 10 × 0.45 NA dry objective (Carl Zeiss, Göttingen, Germany). Cells were counted in the imaged flask area (2.3 mm^2^; cell number, range: 72–3154) using ImageJ, version 1.52 h (Wayne Rasband, National Institutes of Health, Bethesda, MD, USA). Cell proliferation was expressed as an increase in cell number relative to the initial number of cells in culture. Growth rate was calculated based on the following formula:


$$\text{P}={\text{P}}_{0}\times {\text{e}}^{\text{rt}}$$

where P is the cell number at time t, P_0_ is the cell number at time t = 0, e is the Euler’s number (2.71), r is the growth rate, and t is the growth time [100].

### Pulsating fluid shear stress

Cells were seeded at 1.3–2 × 10^3^/cm^2^ on matrigel (Corning)-coated glass slides (2.5 × 6.5 cm), and cultured for 2–4 days. One hour before PFSS, culture media was refreshed by low serum (2% FBS)-containing medium. MuSC cultured on glass slides were subjected to PFSS as described earlier [101]. Briefly, PFSS was generated by pumping 7 ml of culture medium through a parallel-plate flow chamber containing the MuSCs. Cells were subjected to a cyclic changing pressure gradient with a peak shear stress rate of 6.5 Pa/s (pulse amplitude: 1 Pa; pulse frequency: 1 Hz). Static control cells were kept in similar conditions as PFSS-treated cells. After 1 h PFSS treatment or static control culture, images were taken and RNA was isolated. The random images acquired pre-PFSS and post-PFSS were counted to determine the number of young and aged MuSCs detached as a result of PFSS treatment.

### NO analysis

Medium samples were taken from PFSS-treated and static cultures at 10, 30, and 60 min for NO analysis. NO production was measured as nitrite (NO_2_^−^) accumulation in the medium using Griess reagent containing 1% sulfanilamide, 0.1% naphthalene-diamine-dihydrochloride, and 2.5 M H_3_PO_4_. Serial dilutions of NaNO_2_ in medium were used as a standard curve. Absorbance was measured at 540 nm with a microplate reader (BioRad Laboratories Inc., Veenendaal, The Netherlands).

### Immunohistochemistry

To determine MuSC morphology, cells were seeded at 7 × 10^3^/cm^2^ on matrigel (Corning)-coated ibidi μ-Slides (ibidi, Martinsried, Germany), and cultured for 5 days. Cells were washed with PBS and fixed with 4% paraformaldehyde (Thermo-Fisher Scientific, Kandel, Germany) for 10 min. Cells were washed with PBS, and permeabilized for 5 min with 0.5% Triton X-100 in PBS. Cells were washed with PBS, and stained with 100 nM Acti-stain™ 488 phalloidin (Cytoskeleton, Denver, CO, USA) and 100 nM 2-(4-Amidinophenyl)-6-indolecarbamidine dihydrochloride (DAPI; Sigma-Aldrich) for 30 min.

To stain the MuSC glycocalyx, cells were seeded on matrigel-coated ibidi μ-Slides at 7 × 10^3^ cells/cm^2^ or 14 × 10^3^ cells/cm^2^, and cultured for 1 and 3 days. Hyaluronan was stained to determine the presence of a glycocalyx. Cells were washed three times with PBS, and fixed with 2% paraformaldehyde and 0.1% glutaraldehyde (Sigma-Aldrich) for 30 min at room temperature. MuSCs were washed three times with PBS, and blocked with 2% goat serum (Thermo-Fisher Scientific) for 30 min at room temperature, followed by overnight incubation with 2 μg/ml biotinylated hyaluronic acid-binding protein (EMD Millipore, Billerica, MA, USA). MuSCs were then incubated with Alexa Flour 488 conjugated IgG monoclonal anti-Biotin (1/50; Jackson ImmunoResearch Lab, Philadelphia, PA, USA) and Dapi (100ng/mL; Sigma-Aldrich) for 30 min at room temperature, followed by washing three times with PBS.

To stain integrin-α7 (ITGA7), pPXN, and YAP, MuSCs were seeded at 3–13 × 10^3^ cells/cm^2^ on matrigel-(Corning)-coated glass slides (22 × 22 mm), and cultured for 2–4 days. After 30 min of PFSS application the MuSCs were washed twice with pre-warmed (37ºC) PBS and fixed in 4% paraformaldehyde for 10 min. Cells were permeabilized with 0.1% Triton X-100 for 10 min and blocked with 5% goat serum for 60 min. Subsequently, MuSCs were incubated with mouse anti-integrin α7 K0046-3 (1/50; MBL, Woburn, MA, USA) and rabbit anti-Phospho-Paxillin (Tyr31) 44-720G (1/100; Thermo-Fisher Scientific) primary antibodies in 5% goat serum for 1 h at room temperature. After washing three times with PBS, the cells were incubated with anti-rabbit STAR 580, anti-mouse STAR 635 (1/100; Abberior, Göttingen, Germany) secondary antibodies in 5% goat serum for 1 h at room temperature. To stain YAP, MuSCs were incubated with mouse anti-YAP sc-101199 (1/100; Santa Cruz Biotechnology, Dallas, TX, USA) primary antibody, and anti-mouse Alexa Fluor 555 A-21422 (1/500; Thermo-Fisher Scientific) secondary antibody, following the same steps. To stain the nucleus and F-actin, cells were washed three times with PBS, and incubated with DAPI and Acti-stain 488 Phalloidin PHDG 1 (Cytoskeleton, Denver, CO, USA).

### Image acquisition

All images were acquired using the Leica TCS SP8 confocal microscope (Leica Microsystems, Wetzlar, Germany). For imaging myoblast hyaluronan and nuclei, cells were irradiated with a pulsed white light laser at a wavelength of 488 nm and 405 nm, respectively. Cross-sectional images (XZ) and Z-stacks were acquired using 100× 1.4 NA oil objective with a pinhole of 1 airy unit. To image F-actin, integrin-α7, pPXN, and YAP, cells were irradiated with a wavelength of 499 nm, 633 nm, 587 nm, and 554 nm, respectively. To determine cell morphology, young and aged MuSCs were stained for F-actin and nucleus, and irradiated with a pulsed white light laser at a wavelength of 499 nm and 405 nm, respectively. XZ-images and Z-stacks were acquired using 40× 1.3 NA oil objective. The z-distance between images was 150 nm.

### Image analysis

Images were analyzed using ImageJ and cell area was defined by setting a threshold for the F-actin staining. Integrated pixel density of integrin-α7 was determined from the maximum projection image within the cell area. Total number of pPXN clusters per cell and surface area of each cluster were quantified with an adapted version of the focal adhesion quantification method described earlier [102]. The background intensity was subtracted from the raw image, and maximum intensity projection was applied to the z-stacks. Local contrast was enhanced and LoG 3D filter applied, and the threshold was set based on the maximum pixel intensity of the staining. The number and size of the pPXN clusters was expressed as the number and area of the particles in thresholded images.

Integrated pixel density of YAP was determined from the maximum projection images within the cell area. To quantify the nuclear-to-cytoplasmic YAP ratio, the nuclear domain and cytoplasmic domain were segmented from the maximum projection z-stacks. The cytoplasmic domain was computed as the difference between the circumference of the membrane and the nucleus. Integrated pixel density of YAP was quantified in the nuclear area and in the cytoplasmic area.

Analysis of cellular parameters, i.e., cell area, cell volume, nuclear volume, cell aspect-ratio (major-axis/minor-axis), cell roundness 4 × ([area])/(π × [major-axis]^2^), and cell circularity 4π × ([area])/([perimeter]^2^) was performed in ImageJ. Cell apex-height was defined as the distance between the glass surface and the cell apex, and was measured from the XZ images using Leica Application Suite X (LasX; Mannheim, Germany). Z-stack images were thresholded and cells segmented using ImageJ. Cell parameters were determined from the cell basal area attached to the substrate. Segmented cell and nuclear area through the z-stack was used to measure the volume by using ImageJ macro (measure stack).

### MuSC stiffness measurement

MuSCs where seeded at 16 × 10^3^/cm^2^ on matrigel-coated ibidi μ-Slides, and cultured for 1 day using the culture conditions mentioned above. Indentation experiments were conducted at 23°C, in HEPES (Sigma-Aldrich) buffered DMEM based growth medium. Cell stiffness was measured using Chiaro nano-indenter with PIUMA controller (Optics 11, Amsterdam, The Netherlands). A spherical tip (diameter: 7 μm) was used to indent MuSCs at the cell apex by 2.7 μm at a rate of 0.63 μm/s. The Young's modulus was calculated from the first 2 μm of the load-displacement curves using the Hertz spherical indentation model [103]. Before and at maximal indented state, z-stacks were acquired to visualize the cell deformation.

### Fluid shear stress and live cell imaging of MuSC deformation

MuSCs were seeded at 4–17 × 10^3^/cm^2^ on matrigel (Corning)-coated glass slides (22 × 22 mm) and cultured for 1–5 days. Three to 5 h before fluid shear stress application, culture medium was refreshed by medium with low serum (2% FBS) containing 250 nM live cell stains for F-actin (SiR-Actin, Spirochrome, Stein am Rhein, Switzerland) and nucleic acid (SiR700-DNA, Spirochrome). To determine the effect of fluid shear stress on MuSC morphology, the glass slide was placed into a microfluidic chamber connected to a pump. MuSCs were then subjected to CFSS for 2 min by pumping 7 ml medium with a shear stress of 1 Pa/s, and imaged using Leica TCS SP8 confocal microscope as described [20]. Cells were irradiated with a pulsed white light laser at a wavelength of 645 nm, and XZ time-lapse images were acquired using 40× 1.3 NA oil objective. The effect of CFSS on cell apex-height was quantified during the initial 5 sec of CFSS application.

### RNA isolation and reverse transcription

Cells were lysed with 700 μl Trizol (Thermo-Fisher Scientific) and stored at −80°C overnight. Total RNA was isolated using RiboPure™ Kit (Applied Biosystems, Foster City, CA, USA) and quantified (NanoDrop Technologies, Thermo-Fisher Scientific). mRNA (200 ng) was reverse-transcribed to complementary DNA (cDNA) using a High Capacity RNA-to-cDNA kit SuperScript™ VILO™ Mastermix (Applied Biosystems).

### Quantitative Real-Time PCR

Real-Time PCR was performed using the StepOne™ Real-Time PCR system (Applied Biosystems). Primers were designed using Universal Probe library from Roche Diagnostics. Data were analyzed using StepOne™ v2.0 software (Applied Biosystems) and normalized for *18S* ribosomal RNA levels. *18S*, *Ptk2*, *Yap*, *Tead1*, *Tead2*, *Tead3*, *Tead4*, *c-fos*, *Cdkn2a*, *Itga5*, *Itgb5*, *Itga7*, *Ccnd1*, *Cdk4*, *Timp1*, *Ctgf*, *Cyr61*, *Pax7*, *MyoD*, and *Myog* gene expression was measured using SYBR^®^ green (Thermo-Fisher Scientific). Primer sequences are provided in Table 1. *IL-6* gene expression was measured using Taqman^®^ qPCR and inventoried Taqman^®^ gene expression assays (Applied Biosystems).

**Table 1:** Primer sequences used for real-time PCR.

**Symbol**	**Forward primer**	**Reverse primer**
*18S*	GTAACCCGTTGAACCCCATT	CCATCCAATCGGTAGTAGCG
*Itga7*	GACCCCAGAGCTGGCTG	TCAGGGGACAAGCAAAGAGG
*Itgb5*	GCCCGTTATGAAATGGCCTCA	AGCTAGCGTGAGCAAATGGT
*Itga5*	TGCAGTGGTTCGGAGCAAC	TTTTCTGTGCGCCAGCTATAC
*Ccnd1*	TCAAGTGTGACCCGGACTG	GCCTTGGGGTCGACGTT
*Cdk4*	GGGGAAAATCTTTGATCTCATTGGA	AAGGCTCCTCGAGGTAGAGATA
*Timp1*	ATCACGGGCCGCCTAAG	GAAAGCTCTTTGCTGAGCAGG
*Cyr61*	AGAGGCTTCCTGTCTTTGGC	CCAAGACGTGGTCTGAACGA
*MyoD*	AGCACTACAGTGGCGACTCA	GCTCCACTATGCTGGACAGG
*Myog*	CCCAACCCAGGAGATCATTT	GTCTGGGAAGGCAACAGACA
*Ptk2*	GCTTGGACCTGGCATCTTTG	GCAGCAATGTCCCTGTGAAC
*Yap*	CCATGACTCAGGATGGAGAAGT	CTCTGGTTCATGGCAAAACGA
*Tead1*	GAGCGACTCGGCAGATAAGC	CCACACGGCGGATAGATAGC
*Tead2*	CCCGACATTGAGCAGAGTTTT	CCGGCCATACATCTTGCCC
*Tead3*	CAACCAGCACAATAGCGTCCA	CTGAAAGCTCTGCTCGATGTC
*Tead4*	TCCGCCAAATCTATGACAAGTTC	CGATGTTGGTATTGAGGTCTGC
*c-fos*	TCACCCTGCCCCTTCTCA	CTGATGCTCTTGACTGGCTCC
*Cdkn2a*	GATTCAGGTGATGATGATGGGC	GGAGAAGGTAGTGGGGTCCT
*Ctgf*	CCACCCGAGTTACCAATGAC	GCTTGGCGATTTTAGGTGTC
*Pax7*	TCCATCAAGCCAGGAGACA	AGGAAGAAGTCCCAGCACAG

### Statistical analysis

Statistical analysis was performed using IBM SPSS version 24 (SPSS Inc, Chicago, IL, USA). Regression analysis was conducted using SigmaPlot version 12.5 (Systat Software Inc, San Jose, CA, USA). Data were tested for normal distribution, and data transformation was performed if the data was not normally distributed. The three-way repeated measures ANOVA followed by Bonferroni multiple-comparison test was used to test statistically significant differences in MuSC proliferation, growth rate, and NO production data. Differences in cell morphology data were tested using independent-samples *t*-test and Mann-Whitney u test. Linear regression was performed to test the relation between cell morphological properties. The two-way repeated measures ANOVA followed by Bonferroni multiple-comparison test were used to test differences in the cell deformation data and glycocalyx expression. Differences in integrin data was tested by one-way ANOVA followed by Bonferroni multiple-comparison test. Independent-samples Mann-Whitney u test was used to test the pPXN clusters number, pPXN cluster area, and YAP data. The relation between ITGA7 fluorescent intensity, pPXN cluster number/area, YAP fluorescence intensity, and cell area were determined by linear regression. Independent-samples *t*-test and one-way ANOVA followed by Bonferroni multiple-comparison test and independent-samples Mann-Whitney u test were performed for statistical analysis of gene expression data. One-sample and independent-samples *t*-test were used to test cell detachment data and PFSS-induced gene expression data. Data were expressed as mean ± SEM, and *p* < 0.05 was considered significant. For the growth rate data, a *p* < 0.1 was considered significant.
